# Narcolepsy Following Yellow Fever Vaccination: A Case Report

**DOI:** 10.3389/fneur.2016.00130

**Published:** 2016-08-10

**Authors:** Richard E. Rosch, Michael Farquhar, Paul Gringras, Deb K. Pal

**Affiliations:** ^1^Wellcome Trust Centre for Neuroimaging, Institute of Neurology, University College London, London, UK; ^2^Centre for Developmental Cognitive Neuroscience, Institute of Child Health, University College London, London, UK; ^3^Children’s Sleep Medicine Department, Evelina London Children’s Hospital, Guy’s and St. Thomas’ NHS Foundation Trust, London, UK; ^4^Department of Basic and Clinical Neurosciences, Institute of Psychiatry, Psychology and Neuroscience, King’s College London, London, UK; ^5^Department of Paediatric Neurology, Evelina London Children’s Hospital, Guy’s and St. Thomas’ NHS Foundation Trust, London, UK

**Keywords:** sleep disorders, molecular immunogene, HLA, pediatric neurology

## Abstract

Narcolepsy with cataplexy is a rare, but important differential diagnosis for daytime sleepiness and atonic paroxysms in an adolescent. A recent increase in incidence in the pediatric age group probably linked to the use of the Pandemrix influenza vaccine in 2009, has increased awareness that different environmental factors can “trigger” narcolepsy with cataplexy in a genetically susceptible population. Here, we describe the case of a 13-year-old boy with narcolepsy following yellow fever vaccination. He carries the HLA DQB1*0602 haplotype strongly associated with narcolepsy and cataplexy. Polysomnography showed rapid sleep onset with rapid eye movement (REM) latency of 47 min, significant sleep fragmentation and a mean sleep latency of 1.6 min with sleep onset REM in four out of four nap periods. Together with the clinical history, these findings are diagnostic of narcolepsy type 1. The envelope protein E of the yellow fever vaccine strain 17D has significant amino acid sequence overlap with both hypocretin and the hypocretin receptor 2 receptors in protein regions that are predicted to act as epitopes for antibody production. These findings raise the question whether the yellow fever vaccine strain may, through a potential molecular mimicry mechanism, be another infectious trigger for this neuro-immunological disorder.

## Introduction

Narcolepsy is a rare, lifelong sleep disorder that is usually diagnosed in adolescence and early adulthood. It is classified as narcolepsy type 1 vs. narcolepsy type 2 according to the presence or absence of cataplexy and cerebrospinal fluid (CSF) hypocretin deficiency (1). While the exact etiology is unclear, narcolepsy with cataplexy is strongly associated with a significant loss of hypocretin-secreting neurons in the lateral hypothalamus alongside reduced CSF hypocretin-1 levels. Furthermore, there is a well-documented association with HLA DQB1*0602, suggesting a possible autoimmune etiology (2). Here, we present a patient from our tertiary pediatric clinical service, who has a clear history of narcolepsy with cataplexy following recent yellow fever vaccination.

A recent increase in reported cases of narcolepsy with cataplexy has been associated both with the use of the ASO3-conjugated split-virion *H1N1* vaccine Pandemrix in 2009 (3), and with *H1N1* infection (4). Influenza nucleoprotein antibodies have been shown to cross-react with hypocretin receptors (HCRTR1, HCRTR2), suggesting a form of molecular mimicry as the cause for the hypocretin neuron loss (5). Most patients with this “post-vaccination” narcolepsy develop symptoms at a younger than usual age with abrupt onset of cataplexy shortly after the vaccination (mean interval 7 weeks), resulting in an increase of narcolepsy incidence in the pediatric age group in recent years.

## Case Report

A 13-year-old boy attended pediatric neurology clinic with a 2-year history of repeated paroxysmal muscle tone loss, in order to be assessed for possible atonic seizures. Having been previously neurologically intact, symptoms started with an episode in a restaurant where he abruptly lost axial tone and his head hit the table. He subsequently experienced frequent similar episodes, especially when experiencing positive emotions, during laughter or while playing games with excitement. Episodes were varied as follows: they ranged from subtle involuntary head nods, which he could conceal and were not always noticed by others, to complete postural collapse. They were consistently symmetrical, involving both the left and right side of his body. All instances were described as short-lived, lasting no longer than a few seconds.

During the same period, he would drift off to sleep several times a day without warning, and not wake up until roused. At night, his sleep was interrupted, waking up frightened while feeling unable to move. In addition, his mother also noticed a marked weight gain associated with a significant increase in his appetite. He was previously well, with an unremarkable birth and family history. He never received the Pandemrix influenza vaccine, but ~2 weeks prior to symptom onset had been vaccinated with the Stamaril live, attenuated yellow fever vaccine for a family trip to Africa.

**Figure 1:**
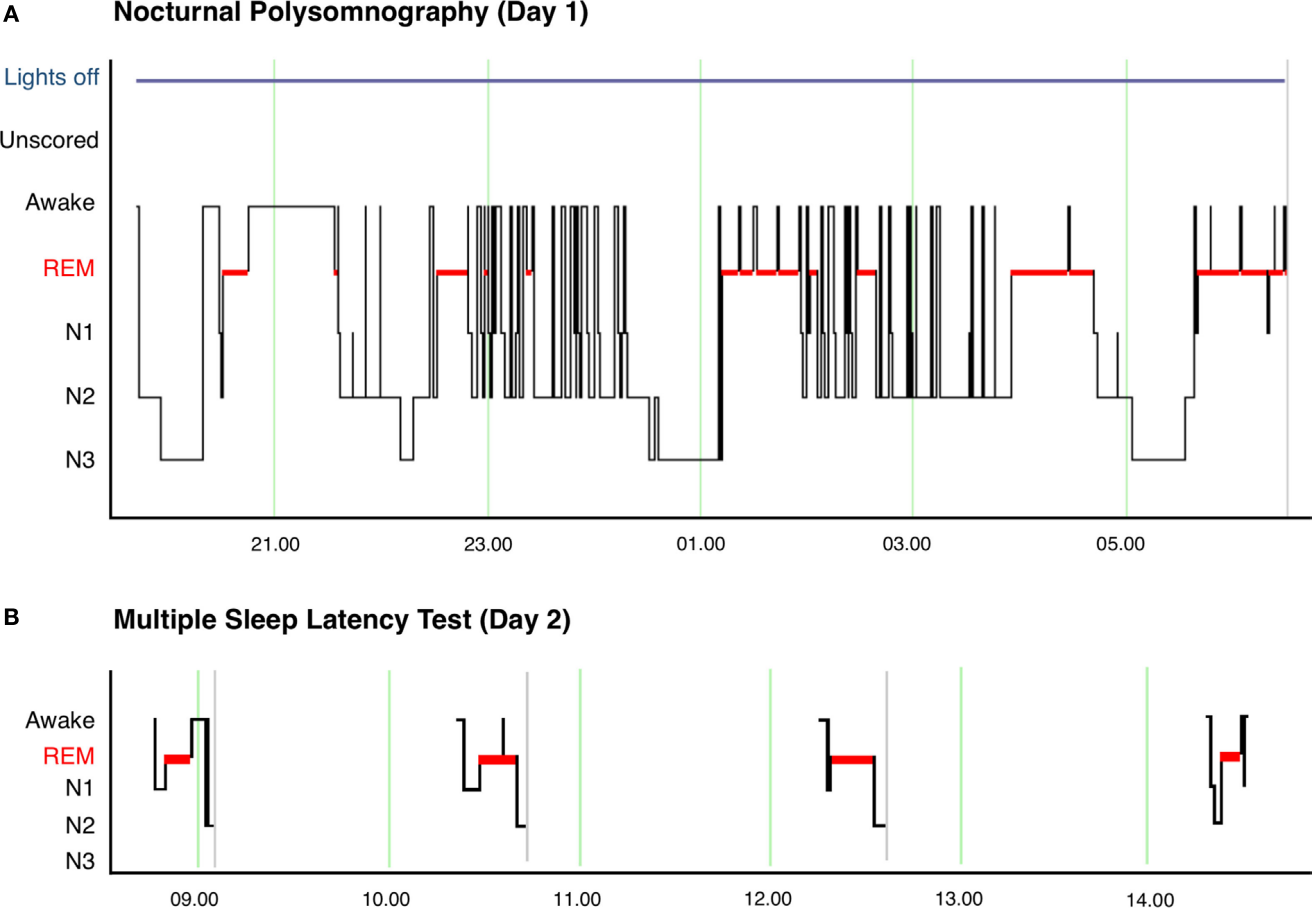
**(A)** Hypnogram of nocturnal polysomnography for patient showing significant sleep fragmentation with frequent arousals and awakenings, particularly during N2 sleep. The image also shows the short sleep latency and frequent sleep onset REM episodes after awakenings. **(B)** Hypnogram of multiple sleep latency showing sleep onset REM in 4 of 4 nap periods.

Neurological clinical examination was unremarkable and initial investigations, including electrocardiography (ECG), brain magnetic resonance imaging (MRI), and routine blood tests were within normal limits. Polysomnography (PSG) showed rapid sleep onset with rapid eye movement (REM) latency of 47 min (normal 70–110, full results given in Table 1, hypnogram of overnight sleep and multiple sleep latency is shown in Figure 1). He had significant sleep fragmentation, with frequent REM intrusions interrupting the usual consolidation of non-REM periods but no evidence of sleep disordered breathing. Multiple sleep latency test (MSLT) performed the following day was significantly abnormal with a mean sleep latency of 1.6 min (normal >8 min) and sleep onset REM evident in four out of four nap periods.

**Table 1:** **Polysomnography results of the patient at time of diagnosis**.

Measurement	Value	Reference
**Nocturnal polysomnography**
Total sleep time, min	646.6	–
Sleep onset latency, min	0.7 (rapid)	23 (SD = 25)
REM latency, min	47 (rapid)	88 (SD = 41)
Arousal index, per hour	16.4 (slightly elevated)	9.3 (SD = 4.8)
Sleep efficiency	82.5% (slightly low)	89 (SD = 7.5)
Oxygen saturation, %	98.1	>96
Apnea hypopnea index, per hour	1.5	1 (SD = 0.8)
**Sleep stages, % of total sleep time**		
N1	5.9	~6^b^
N2	41.1	~49^b^
N3	18.5	~23^b^
REM	34.5	~22^b^
**Multiple sleep latency test**
Mean sleep latency test, min	1.6 (rapid)	>8^a^
Sleep onset REM periods	4 of 4 nap periods (suggestive of narcolepsy)	0^a^

*^a^Indicates significantly abnormal results*.

*^b^Reference values from Ref. (6) given for comparison, no confidence intervals given*.

*REM, rapid eye movement*.

The clinical history of weight gain, hypnagogic hallucinations, sleep paralysis, and clear episodes of loss of muscle tone (cataplexy) is strongly suggestive of narcolepsy. With that clinical context, the fragmented sleep without sleep disordered breathing on PSG together with significantly shortened sleep latency and sleep onset REM on MSLT confirmed the diagnosis of narcolepsy. Periods of non-REM sleep were interrupted by REM intrusions, resulting in a proportional increase in REM sleep compared to non-REM sleep phases (as indicated in Table 1). While sleep fragmentation does occur for other reasons, this pattern shown on PSG is typical of young patients with untreated narcolepsy. These findings taken together meet level 2 evidence for narcolepsy according to the criteria laid out by the Brighton Collaboration Narcolepsy Working Group (7), as well as fulfilling the diagnostic criteria for narcolepsy type 1 defined in the International Classification of Sleep Disorders, third edition (1).

He was found to have the strongly associated HLA-type DQB1*0602. MRI brain, including T1 and T2 weighted scan and FLAIR imaging was normal, with normal hypothalamic appearances. Because of the clear clinical history of cataplexy and the invasiveness of the procedure in a pediatric patient, CSF levels of hypocretin (orexin) were not measured. Treatment was mainly directed at managing daytime sleepiness through improved sleep hygiene and daytime naps, in addition to stimulant medication (slow release methylphenidate, 18 mg once a day increased in two steps to 45 mg once a day) and anti-cataplectic medication (venlafaxine, 37.5 mg once a day).

## Discussion

The case presented here raises the possibility that currently unknown combinations of genetic susceptibilities and environmental triggers may play a role in the etiology of narcolepsy in some patients. Our patient never received Pandemrix, while having received the yellow fever vaccine Stamaril 2 weeks prior to cataplexy onset. In cases reported in association with Pandemrix, the risk of developing narcolepsy was most increased in this early post-vaccination time period (8–42 days) (8). The yellow fever vaccines induce a strong immune response, and the YFV-17D strain used in the Stamaril vaccines contains multiple antigens with several epitopes each that can bind different HLA molecules (9). While yellow-fever-vaccine-associated neurological disease is described, narcolepsy is not currently a recognized complication (10).

Previous studies have shown that the association with HLA DQB1*0602 is extremely high in patients with typical or severe cataplexy [85–95% of patients show this HLA type vs. 25% of age-matched controls (11)]. Together with epidemiological associations between narcolepsy onset and different environmental triggers [including *Streptococcus sp*. Infection (12), *H1N1* infection (13), and the Pandemrix vaccine (7)], an immune-mediated, environmentally triggered etiology appears most likely (14). Most recently, different lines of evidence have suggested that the *H1N1*- and Pandemrix-associated cases of narcolepsy are caused by molecular mimicry between influenza epitopes and hypocretin receptor domains with associated antibody-mediated destruction of hypocretin-secreting neurons (5).

The attenuated yellow fever strain used in the Stamaril vaccine (YFV-17D) carries a genome that codes for 13 distinct proteins and peptides [UniProt ID P03314 (15)]. Amino acid sequences for each of the 17D strain peptides and proteins were assessed for sequence overlap with hypocretin, HCRTR1, and HCRTR2 using the BLAST algorithm (see blast.ncbi.nlm.nih.gov for more information). Of these, four show significant amino acid sequence similarities to hypocretin or one of its receptors (Table 2).

**Table 2:** **Sequence overlap between YFV-D17 proteins and hypocretin and its receptors**.

Protein/peptide	AA#	Reference sequence	AA#	Cellular location	*E*-value
Capsid protein C	2–13	Hypocretin receptor 1	157–168	Cytoplasmic/transmemrbrane	0.14
Capsid protein C	65–74	Hypocretin receptor 2	262–271	Cytoplasmic	0.29
Envelope protein E	224–235	Hypocretin receptor 2	43–54	Extracellular	0.47
Envelope protein E	218–253	Hypocretin	70–103	Extracellular	0.055
Non-structural protein 2A-alpha	128–152	Hypocretin receptor 2	137–161	Cytoplasmic/transmembrane	0.63
Serine protease subunit NS2B	95–108	Hypocretin receptor 2	422–435	Cytoplasmic	0.11

*AA#, position in the amino acid sequence (start–stop); *E*-value, “Expect” value is a parameter that describes the number of hits “expected” chance results; the lower the score, the more “significant” the match is (see http://blast.ncbi.nlm.nih.gov/ for more information)*.

Sequence overlaps in extracellular domains of the human proteins are of particular interest as these are the most plausible target for putative antibody-mediated molecular mimicry. Only one of the viral peptides (the envelope protein E) has sequence overlaps with extracellular protein components (i.e., hypocretin, or external domains of the hypocretin receptors).

**Figure 2:**
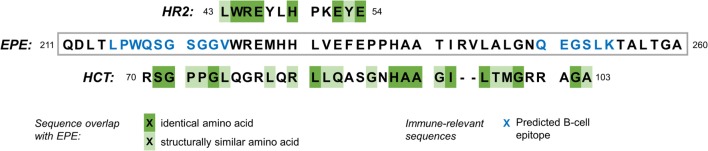
**Amino acid sequence overlap between a segment of the YFV-17D envelope protein E (EPE), the human hypocretin receptor 2 (HR2, top), and hypocretin (HCT, bottom)**. Blue letters in the EPE sequence show amino acids that are predicted to constitute an epitope for B-cells [BepiPred Linear Epitope Prediction, see tools.immuneepitope.org (16)]. Amino acids are grouped into six groups according to a composite difference score calculated from polarity, volume, and composition. They are classified as similar where they belong to the same group (17).

Sections within the same protein sequence are also predicted to act as epitopes for B-cell linear proliferation (Figure 2). These findings taken together illustrate one specific possible mechanism for molecular mimicry between the yellow fever vaccine strain, and hypocretin-secreting neurons: antibodies are produced to epitopes found in the envelope protein E sequence as part of the strong immune response to the vaccine. These may then cross-react with either hypocretin molecules themselves, or an extracellular domain of the hypocretin receptor 2, because of the amino acid sequence similarities between the vaccine epitopes and the hypocretin or receptor molecules.

Clearly, our single case does not allow any inference whether Stamaril has causally contributed to the development of narcolepsy in this patient. Hypocretin-associated autoantibodies, where detected at all, do not seem to be more common in patients with narcolepsy compared to controls (18, 19). And even for the Pandemrix vaccine, where there is strong epidemiological evidence for a causative link, there is conflicting evidence as to whether molecular mimicry plays a role in the pathophysiology (5, 20).

Yet our case raises the consideration as to whether other associations between environmental triggers and narcolepsy onset exist, particularly in the context of the genetic susceptibility conferred by the associated HLA type.

## Conclusion

Narcolepsy with cataplexy in children is strongly associated with HLA DQB1*0602 and a loss of hypothalamic hypocretin-secreting neurons. There is epidemiological evidence that a recent increase in narcolepsy diagnoses in children was linked to the *H1N1* mass vaccination.

The close temporal association of the live-attenuated yellow fever vaccine, associated with genetic susceptibility, and the potential for molecular mimicry between MHC-derived peptides, hypocretin, its receptors, and the viral envelope protein E raises the question as to whether the vaccine was involved in the pathology here. Exploring any such association will need further careful evaluation using epidemiological approaches. The next step for this will be a detailed evaluation of the incidence of narcolepsy in possible at risk population (i.e., patients exposed to yellow fever in endemic regions, young people undergoing yellow fever vaccine). As with the Pandemrix vaccine-related cases, further longitudinal follow-up is necessary, as it is not yet clear whether presumed vaccine-associated cases follow the same natural history as seen in other narcolepsy patients.

## Ethics Statement

No investigations or interventions were performed outside routine clinical care for this patient. As this is a case report, without experimental intervention into routine care, no formal research ethics approval was required; Written, fully informed consent was given and recorded from the patient’s parents. Verbal assent was given by the (underage) patient himself; this case study reports routine clinical care provided for a paediatric patient only.

## Author Contributions

Dr. MF and Prof. DP were involved in the work-up of the patient, planning and conducting investigations, and providing clinical care. They reviewed and revised the manuscript and approved the final manuscript as submitted. Dr. RR planned the case report, drafted the initial manuscript, reviewed and revised the manuscript, and approved the final manuscript as submitted. Prof. PG helped planning clinical investigations, critically reviewed the manuscript, implemented substantial changes to the argument, and approved the final manuscript as submitted.

## Conflict of Interest Statement

The authors declare that the research was conducted in the absence of any commercial or financial relationships that could be construed as a potential conflict of interest.
